# Supplementary material for: PTK2B promotes TBK1 and STING oligomerization and enhances the STING-TBK1 signaling
Source: Nat Commun. 2023 Nov 21;14:7567. doi: 10.1038/s41467-023-43419-4 (PMC10663505; doi:10.1038/s41467-023-43419-4)
Supplement: Supplementary file 1 — Supplementary Information [file 41467_2023_43419_MOESM1_ESM.pdf]

| Protein | No.of Peptides | PSMs |
|---|---|---|
| TBK1 | 472 | 53 |
| HSP90AA1 | 21 | 74 |
| HNRNPA2B1 | 6 | 20 |
| PTK2B | 5 | 11 |
| Cep170 | 4 | 5 |
| FGR | 3 | 12 |
| PRMT1 | 1 | 1 |

**Table S1. Identification of TBK1-interacting proteins by Co-IP coupled with mass spectrometry analysis**

TBK1 was purified using S-protein agarose beads from HEK293T cells expressing S protein-Flag-Streptavidin binding peptide (SFB)-tagged mouse TBK1 or control vector cells, and then the protein was incubated with cell lysates from RAW 264.7 cells, followed by Co-IP assays and mass spectrometry analysis. A couple of the selected TBK1-interacting proteins identified by MS analysis were listed.

Table S2 ASO sequences and modification patterns

| ASO Name | Sequence |
|---|---|
| PTK2B-Control | (MOE-5-Me-C)*(MOE-T)*(MOE-T)*(MOE-T)*(MOE-A)*(dG)*(dG)*(dG) *(dA)* (dG)* (dA)*(dT)*(dG)*(dT)*(dG)*(MOE-G)*(MOE-T)*(MOE-G)* (MOE-5-Me-C)*(MOE-A) |
| PTK2B-1 | (MOE-A)*(MOE-5-Me-C)*(MOE-A)*(MOE-G)*(MOE-A)*(dT)*(dG)*(m5d-C)* (m5dC)* (m5dC)*(dA)*(dG)*(dA)*(dT)*(dG)*(MOE-A)*(MOE-G)*(MO-E-T)* (MOE-5-Me-C)* (MOE-A) |
| PTK2B-2 | (MOE-5-Me-C)*(MOE-T)*(MOE-T)*(MOE-5-Me-C)*(MOE-5-Me-C)*(m5d-C)* (m5dC)* (dA)*(m5dC)*(dG)*(dG)*(m5dC)*(dT)*(dG)*(m5dC)*(MOE-5 -Me-C)* (MOE-T)*(MOE-T)*(MOE-A)*(MOE-T) |

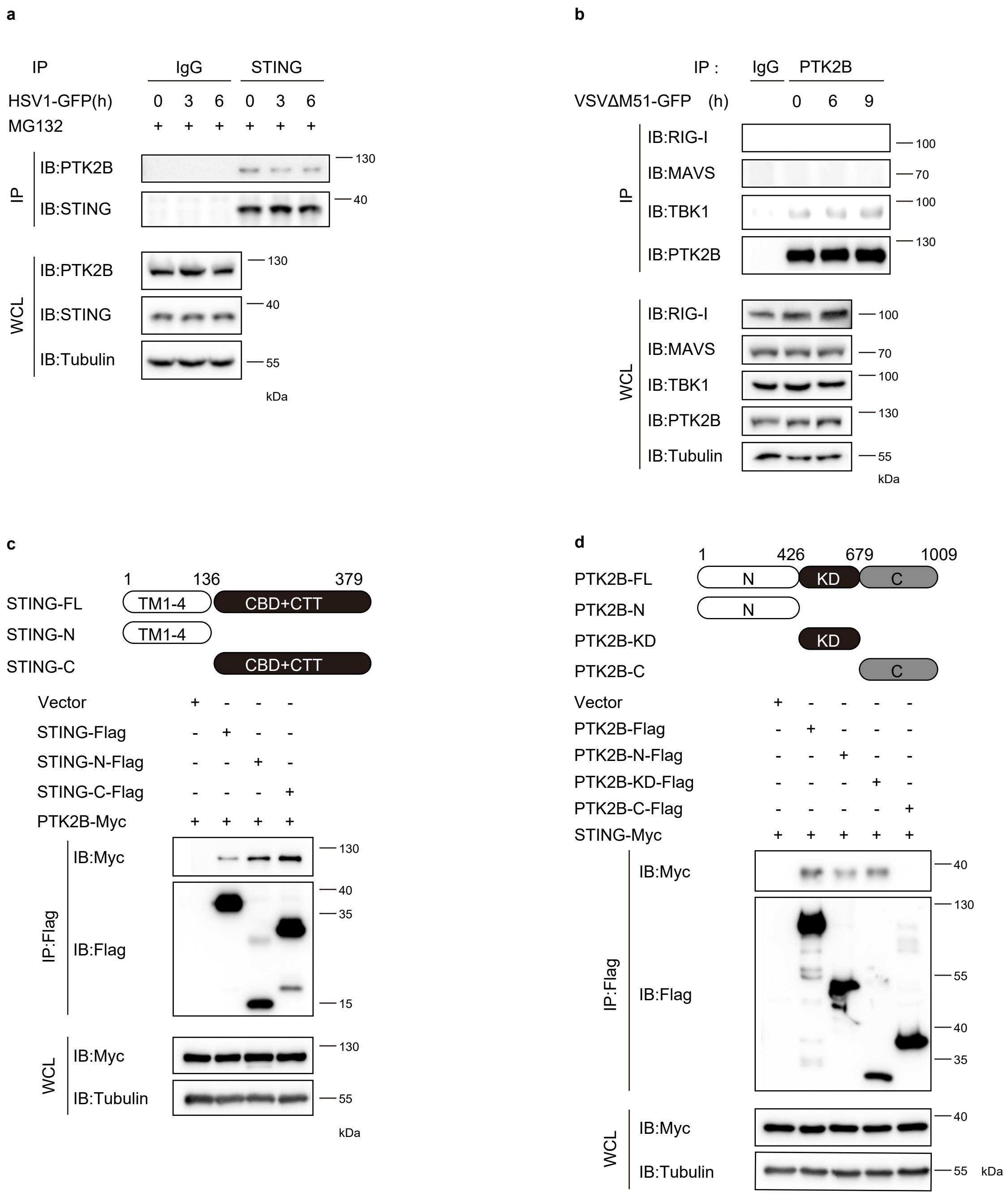


**Fig. S1 PTK2B associates with STING and TBK1, not RIG-I and MAVS**

(a) Raw 264.7 cells were pretreated with MG132 (25uM) for 6 h, then mock infected or infected with HSV1-GFP for the indicated times. The cell lysates were immunoprecipitated with anti-STING antibody or control IgG and analyzed by immunoblotting.

(b) THP1 cells were mock infected or infected with VSVΔM51-GFP for the indicated times. The cell lysates were immunoprecipitated with anti-PTK2B antibody or control IgG and analyzed by immunoblotting.

(c) Schematic diagram of STING domains (top). HEK293T cells were co-transfected with PTK2B-Myc and STING-Flag or its truncated mutants as indicated. Co-IP assays were performed with Flag M2 beads and the pulled-down proteins were analyzed by immunoblotting (bottom).

(d) Schematic diagram of PTK2B domains (top). HEK293T cells were co-transfected with STING-Myc and PTK2B-Flag or its truncated mutants as indicated. Co-IP assays were performed with anti-Flag M2 beads and the pulled-down proteins were analyzed by immunoblotting (bottom).

Data are one representative of two independent experiments with similar results in (a–d). Source data are provided in Fig.S13.

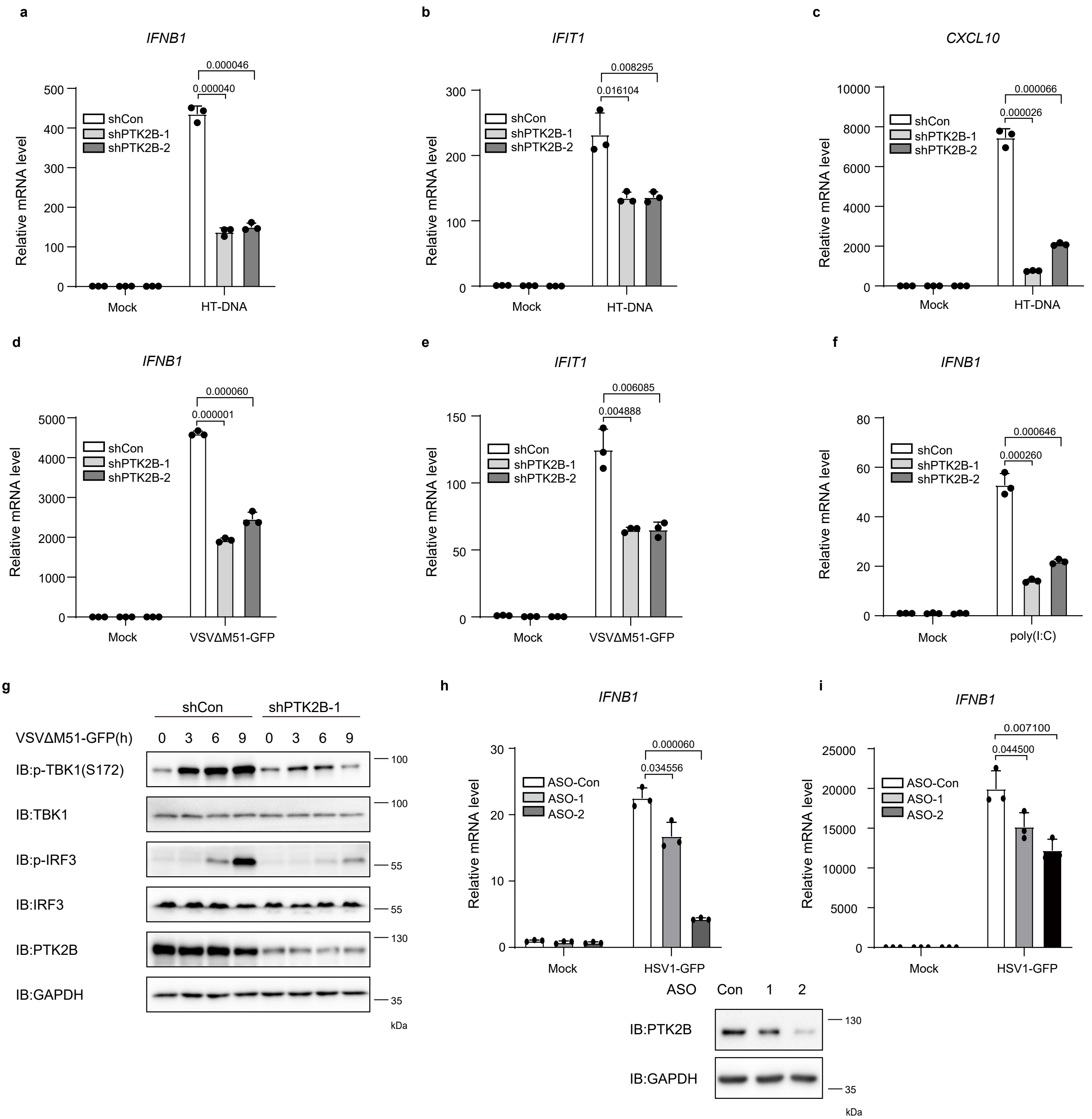


**Fig. S2 Depletion of *PTK2B* attenuates antiviral signaling**

(a–c) THP-1 cells were infected with shRNA lentivirus targeting two different regions of PTK2B (shPTK2B-1, shPTK2B-2) or negative control (shCon), followed by transfection with HT-DNA(1μg/ml) for 6 h. qPCR assays were performed to measure the mRNA levels of *IFNB1* (a), *IFIT1* (b) and *CXCL10* (c).

(d, e) THP1 cells were infected with shRNA lentivirus targeting two different regions of PTK2B(shPTK2B-1, shPTK2B-2) or negative control(shCon), followed by infection with VSVΔM51-GFP for 6 h. qPCR assays were performed to measure mRNA levels of *IFNB1* (d) and *IFIT1* (e).

(f) Similar to (a), except the cells were transfected with poly (I:C).

(g) THP-1 cells stably expressing shRNA targeting PTK2B or control cells were infected with VSVΔM51-GFP for the indicated times, followed by immunoblotting.

(h, i) A549 cells were transfected with ASO-1 or ASO-2 targeting PTK2B, then mock infected or infected with HSV1-GFP (h) for 18 h and VSV-GFP (i) for 6 h. The cells were harvested for qPCR to measure the mRNA levels of *IFNB1*(top) or for immunoblotting with the indicated antibodies(bottom).

Data shown in (a–f, h, i) are from one representative experiment of three independent experiments (mean ± SD, n=3 independent samples), two-tailed Student's t-test. Data are one representative of two independent experiments with similar results in (g). Source data in (a–f, h, i) are provided as a Source Data file, source data in (g) are provided in Fig. S13.

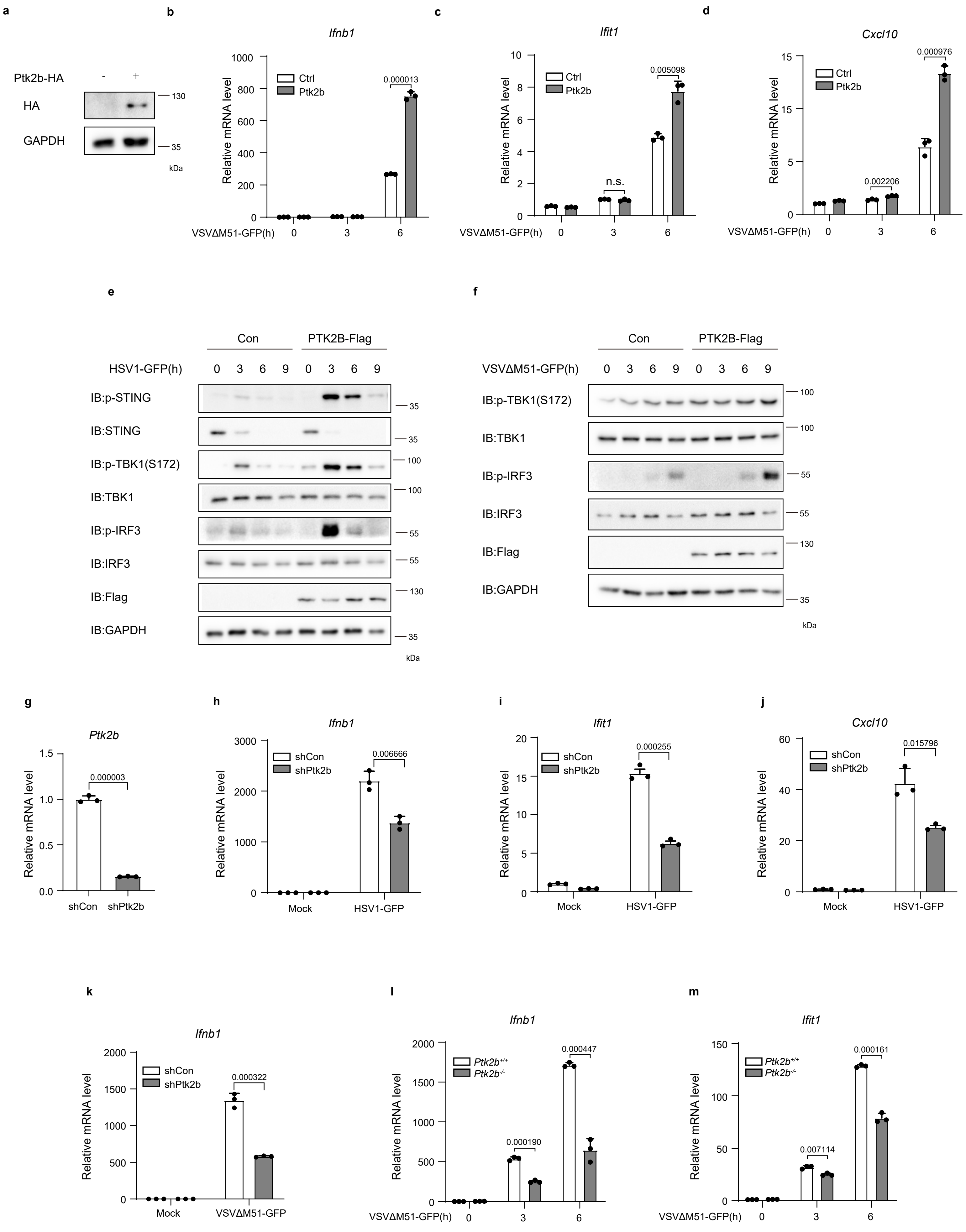

a
Ptk2b-HA
HA
GAPDH
130
35
kDa
b
Ifnb1
Ctrl
Ptk2b
0.000013
Relative mRNA level
VSVΔM51-GFP(h)
c
Ifit1
n.s.
0.005098
d
Cxcl10
0.002206
0.000976
e
Con
PTK2B-Flag
HSV1-GFP(h)
IB:p-STING
IB:STING
IB:p-TBK1(S172)
IB:TBK1
IB:p-IRF3
IB:IRF3
IB:Flag
IB:GAPDH
f
VSVΔM51-GFP(h)
g
Ptk2b
0.000003
shCon
shPtk2b
h
0.006666
Mock
HSV1-GFP
i
0.000255
j
0.015796
k
0.000322
VSVΔM51-GFP
l
Ptk2b+/+
Ptk2b-/-
0.000190
0.000447
m
0.007114
0.000161

**n**

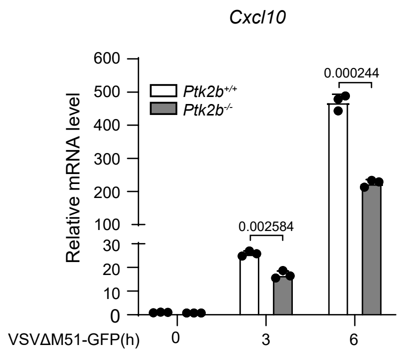


**o**

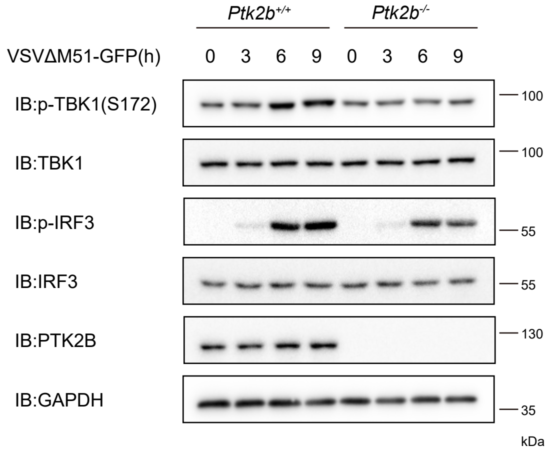


**Fig. S3 Alteration of PTK2B expression affects antiviral signaling in mouse MEFs and Raw264.7 cells**

(a) Immortalized MEFs stably expressing PTK2B or control cells were lysed for immunoblotting.

(b–d) Immortalized MEFs stably expressing PTK2B or control cells were infected with VSVΔM51-GFP for 3 and 6 h. The mRNA levels of *Ifnb1* (b), *Ifit1* (c) and *Cxcl10* (d) were measured by qPCR.

(e, f) Immortalized MEFs stably expressing PTK2B or control cells were infected with HSV1-GFP (e) or VSVΔM51-GFP (f) for the indicated times, followed by immunoblotting with the indicated antibodies.

(g–j) Raw264.7 cells were infected with shRNA lentivirus targeting *Ptk2b* or negative control, followed by infection with HSV1-GFP for 6 h. qPCR assays were performed to measure mRNA levels of *Ptk2b* (g), *Ifnb1* (h), *Ifit1* (i) and *Cxcl10* (j).

(k) Similar to (h), except that VSVΔM51-GFP was used for stimulation.

(l–n) *Ptk2b*$^{+/+}$ and *Ptk2b*$^{-/-}$ RAW cells were infected with VSVΔM51-GFP for 3 and 6 h, and then analyzed by qPCR to quantify mRNA levels of *Ifnb1* (l), *Ifit1* (m) and *Cxcl10* (n).

(o) *Ptk2b*$^{+/+}$ and *Ptk2b*$^{-/-}$ Raw264.7 cells were infected with VSVΔM51-GFP for the indicated times, and then analyzed by immunoblotting with the indicated antibodies.

Data shown in (a–d, g–n) are from one representative experiment of three independent experiments (mean ± SD, n=3 independent samples). n.s. not significant, two-tailed Student's t-test. Data are one representative of two independent experiments with similar results in (e, f, o). Source data in (b-d, g-n) are provided as a Source Data file, source data in (e, f, o) are provided in Fig. S13.

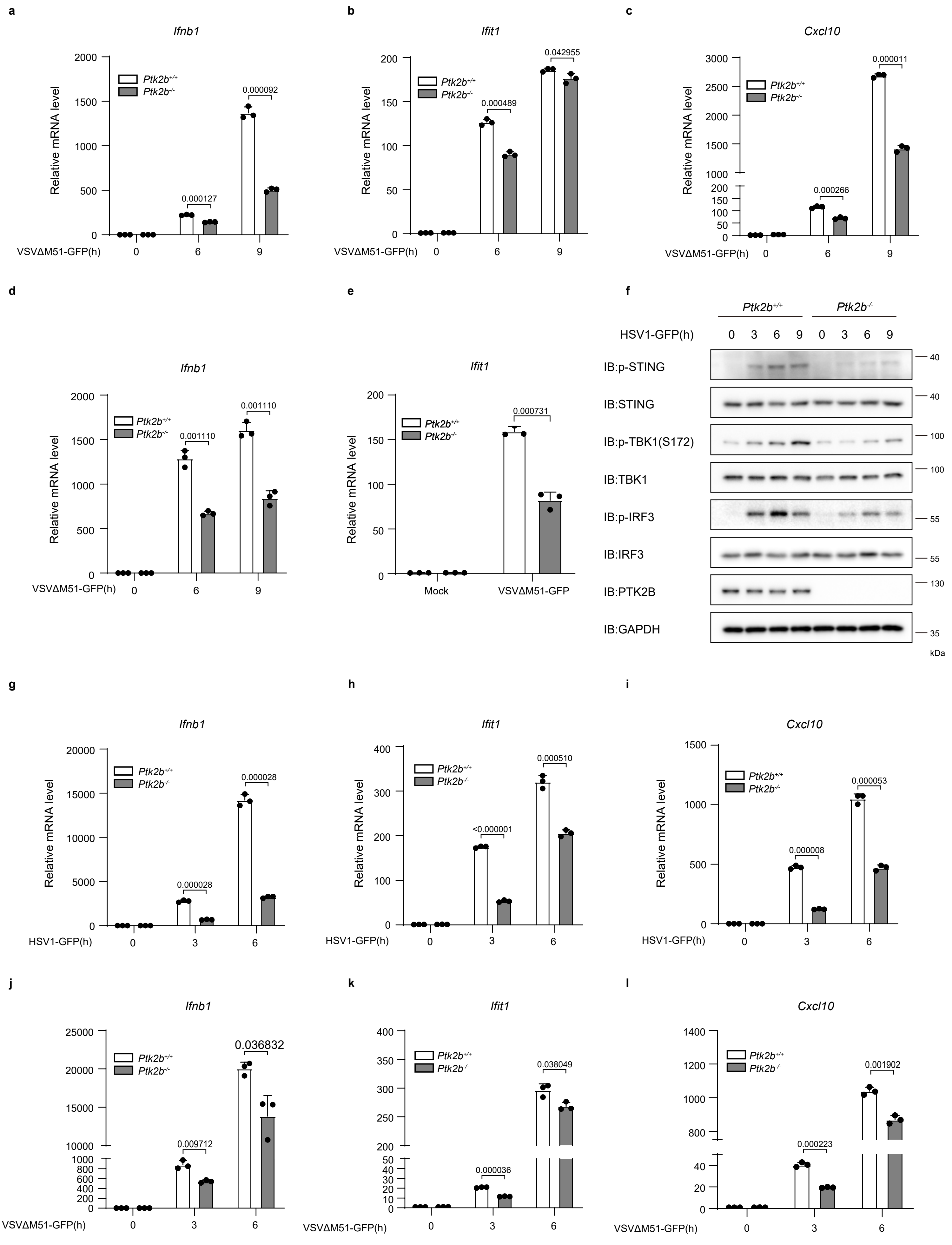
a
Ifnb1
Relative mRNA level
2000
1500
1000
500
0
Ptk2b+/+
Ptk2b-/-
0.000127
0.000092
VSVΔM51-GFP(h)
0
6
9
b
Ifit1
Relative mRNA level
200
150
100
50
0
Ptk2b+/+
Ptk2b-/-
0.000489
0.042955
VSVΔM51-GFP(h)
0
6
9
c
Cxcl10
Relative mRNA level
3000
2500
2000
1500
1000
200
150
100
50
0
Ptk2b+/+
Ptk2b-/-
0.000266
0.000011
VSVΔM51-GFP(h)
0
6
9
d
Ifnb1
Relative mRNA level
2000
1500
1000
500
0
Ptk2b+/+
Ptk2b-/-
0.001110
0.001110
VSVΔM51-GFP(h)
0
6
9
e
Ifit1
Relative mRNA level
200
150
100
50
0
Ptk2b+/+
Ptk2b-/-
0.000731
Mock
VSVΔM51-GFP
f
Ptk2b+/+
Ptk2b-/-
HSV1-GFP(h)
0 3 6 9 0 3 6 9
IB:p-STING
IB:STING
IB:p-TBK1(S172)
IB:TBK1
IB:p-IRF3
IB:IRF3
IB:PTK2B
IB:GAPDH
40
40
100
100
55
55
130
35
kDa
g
Ifnb1
Relative mRNA level
20000
15000
10000
5000
0
Ptk2b+/+
Ptk2b-/-
0.000028
0.000028
HSV1-GFP(h)
0
3
6
h
Ifit1
Relative mRNA level
400
300
200
100
0
Ptk2b+/+
Ptk2b-/-
<0.000001
0.000510
HSV1-GFP(h)
0
3
6
i
Cxcl10
Relative mRNA level
1500
1000
500
0
Ptk2b+/+
Ptk2b-/-
0.000008
0.000053
HSV1-GFP(h)
0
3
6
j
Ifnb1
25000
20000
15000
10000
1000
800
600
400
200
0
Ptk2b+/+
Ptk2b-/-
0.009712
0.036832
VSVΔM51-GFP(h)
0
3
6
k
Ifit1
400
350
300
250
200
50
40
30
20
10
0
Ptk2b+/+
Ptk2b-/-
0.000036
0.038049
VSVΔM51-GFP(h)
0
3
6
l
Cxcl10
1400
1200
1000
800
40
20
0
Ptk2b+/+
Ptk2b-/-
0.000223
0.001902
VSVΔM51-GFP(h)
0
3
6

**Fig. S4 *Ptk2b* knockout reduces antiviral signaling in MEFs, BMDMs and BMDCs**

(a–c) Primary *Ptk2b*$^{+/+}$ and *Ptk2b*$^{-/-}$ MEFs were infected with VSVΔM51-GFP for the indicated times, and then lysed to measure mRNA levels of *Ifnb1* (a), *Ifit1* (b) and *Cxcl10* (c) by qPCR.

(d–e) *Ptk2b*$^{+/+}$ and *Ptk2b*$^{-/-}$ BMDMs were infected with VSVΔM51-GFP for the indicated times, followed by qPCR to measure mRNA levels of *Ifnb1* (d) and *Ifit1* (e).

(f) *Ptk2b*$^{+/+}$ and *Ptk2b*$^{-/-}$ BMDMs were infected with HSV1-GFP for the indicated times, followed by immunoblotting.

(g–i) *Ptk2b*$^{+/+}$ and *Ptk2b*$^{-/-}$ BMDCs were infected with HSV1-GFP for the indicated times, followed by qPCR to measure mRNA levels of *Ifnb1* (g), *Ifit1* (h) and *Cxcl10* (i).

(j–l) *Ptk2b*$^{+/+}$ and *Ptk2b*$^{-/-}$ BMDCs were infected with VSVΔM51-GFP for the indicated times, followed by qPCR to measure mRNA levels of *Ifnb1* (j), *Ifit1* (k) and *Cxcl10* (l).

Data are one representative of two independent experiments with similar results in (f). Data shown in (a–e, g–l) are from one representative experiment of three independent experiments (mean ± SD, n=3 independent samples), two-tailed Student's t-test. Source data in (a–e, g–l) are provided as a Source Data file, source data in (f) is provided in Fig. S13.

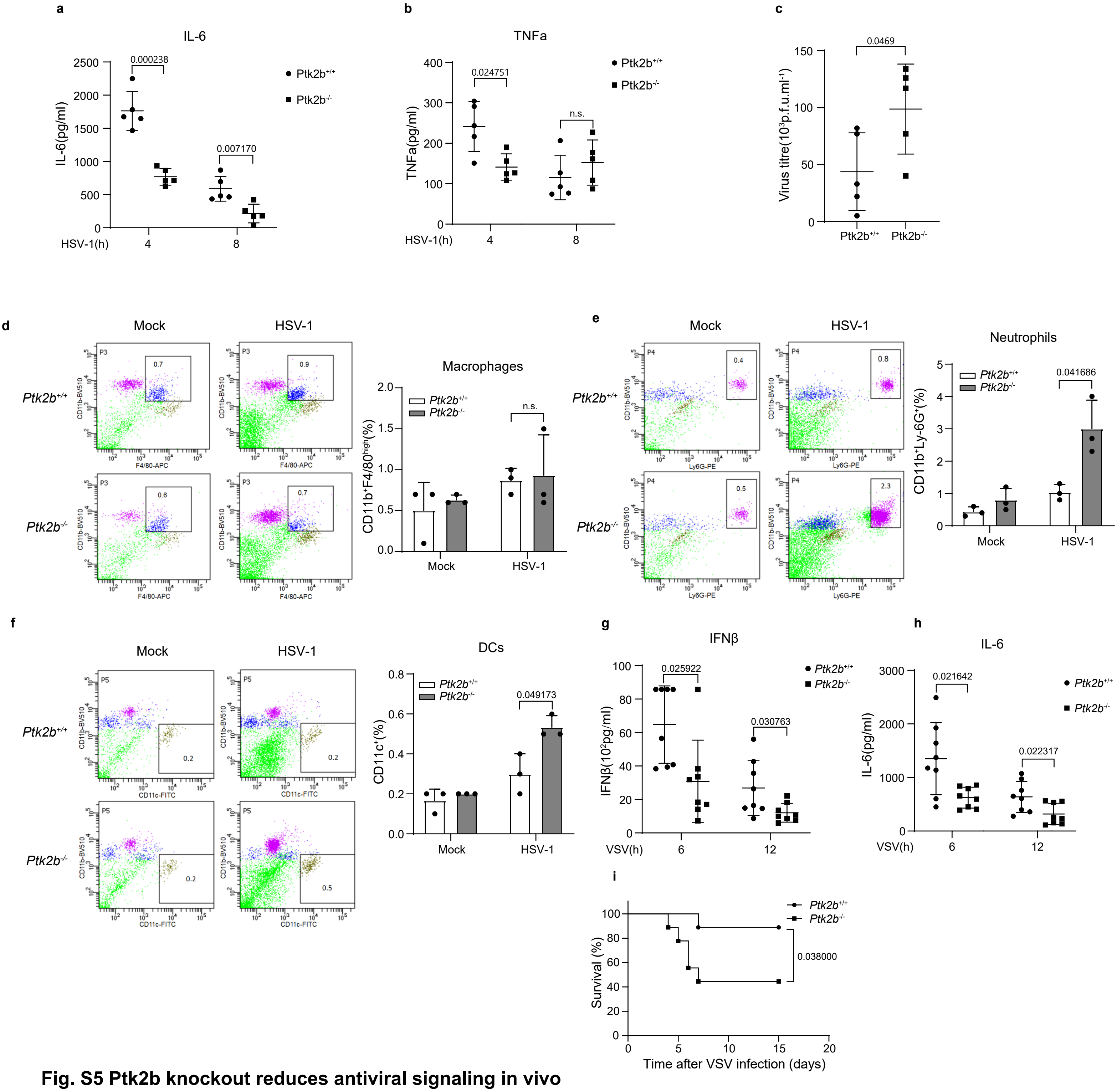


**Fig. S5 Ptk2b knockout reduces antiviral signaling in vivo**

(a, b) *Ptk2b*$^{+/+}$ and *Ptk2b*$^{-/-}$ mice were infected with HSV-1 via tail vein injection at 4×10$^{7}$ pfu per mouse. Sera were collected at 4 and 8 h after infection to measure the levels of IL-6 (a) and TNFα (b) by ELISA. n =5 mice for each group.

(c) *Ptk2b*$^{+/+}$ and *Ptk2b*$^{-/-}$ mice were infected with HSV-1 via tail vein injection at 3x10$^{7}$ pfu per mouse for 4 days. Viral titers in the infected brain were measured using a plaque assay. n =5 mice for each group.

(d–f) *Ptk2b*$^{+/+}$ and *Ptk2b*$^{-/-}$ mice were infected with HSV-1 via tail vein injection at 3x10$^{7}$ pfu per mouse for 4 days. Inflammatory infiltrating cells of lung from mock or infected mice were analyzed using flow cytometry. Representative flow cytometric profiles (left) and frequency (right) of Macrophages (CD11b$^{+}$F4/80$^{high}$) (d), Neutrophils (CD11b$^{+}$Ly-6G$^{+}$) (e) and DCs (CD11c$^{+}$) (f). n =3 mice for each group.

(g, h) *Ptk2b*$^{+/+}$ and *Ptk2b*$^{-/-}$ mice were infected with VSV via tail vein injection at 1×10$^{8}$ pfu per mouse. Sera were collected at 6 and 12 h after infection to measure the levels of IFNβ (g) and IL-6 (h) by ELISA. n =8 mice for each group.

(i) *Ptk2b*$^{+/+}$ and *Ptk2b*$^{-/-}$ mice were infected with VSV via tail vein injection at 1×10$^{8}$ pfu per mouse and the survival of mice was monitored for 15 days. n =9 mice for each group.

Data shown in (a–h) are from one representative experiment of two independent experiments (mean ± SD). n.s. not significant, two-tailed Student's t-test. The log-rank (Mantel–Cox) test was used in Data (i). Source data are provided as a Source Data file.

a

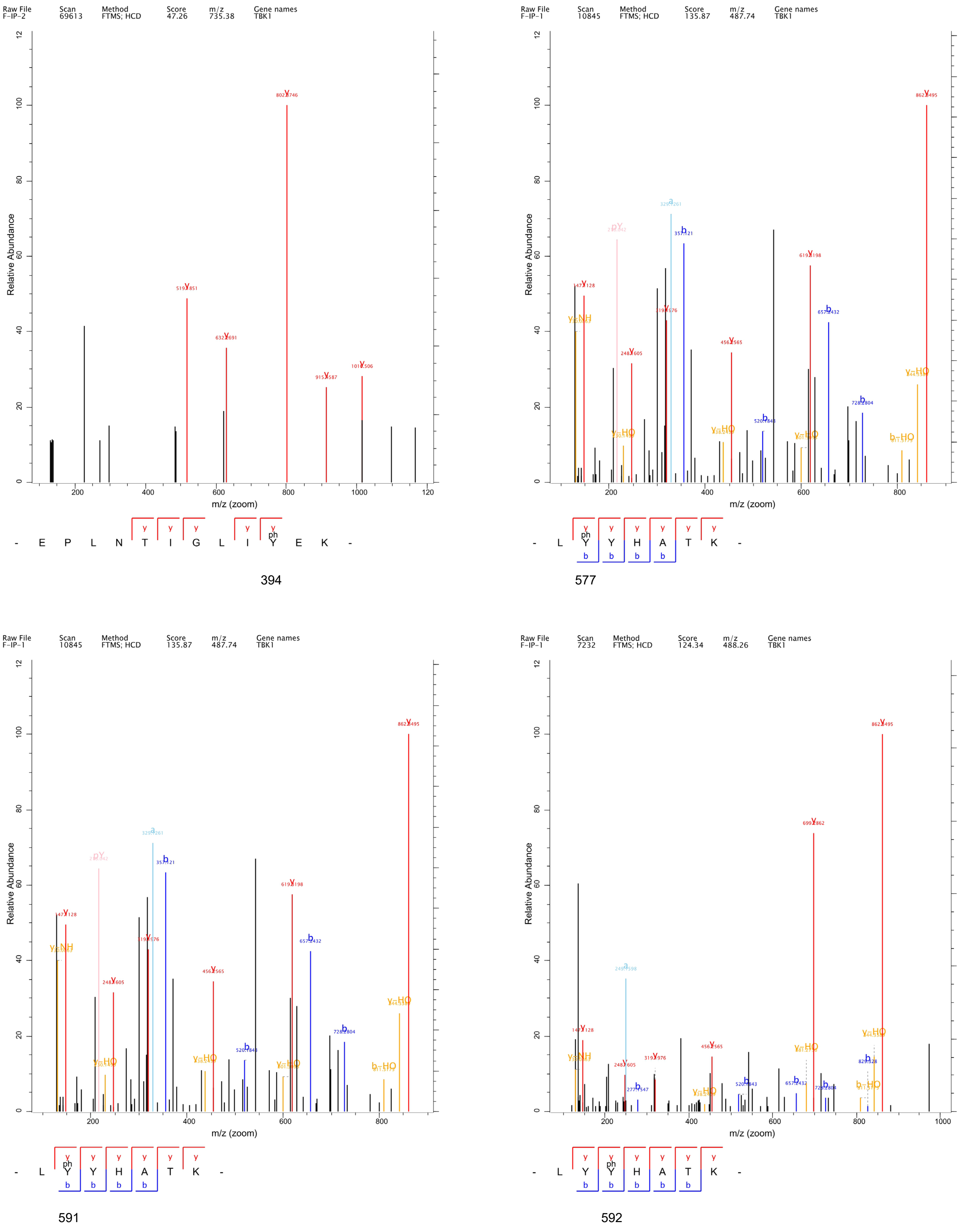


**Fig. S6 Identification of phosphorylated Tyr residues of TBK1 by PTK2B**

(a) Flag-tagged TBK1 was co-transfected into HEK293T cells with Myc-tagged PTK2B or control vector, cell lysates were immunoprecipitated with anti-Flag M2 beads, followed by mass spectrometric analysis. Mass spectrometric analysis identified Tyr residues Y394, Y577, Y591 and Y592 of TBK1 phosphorylated by PTK2B.

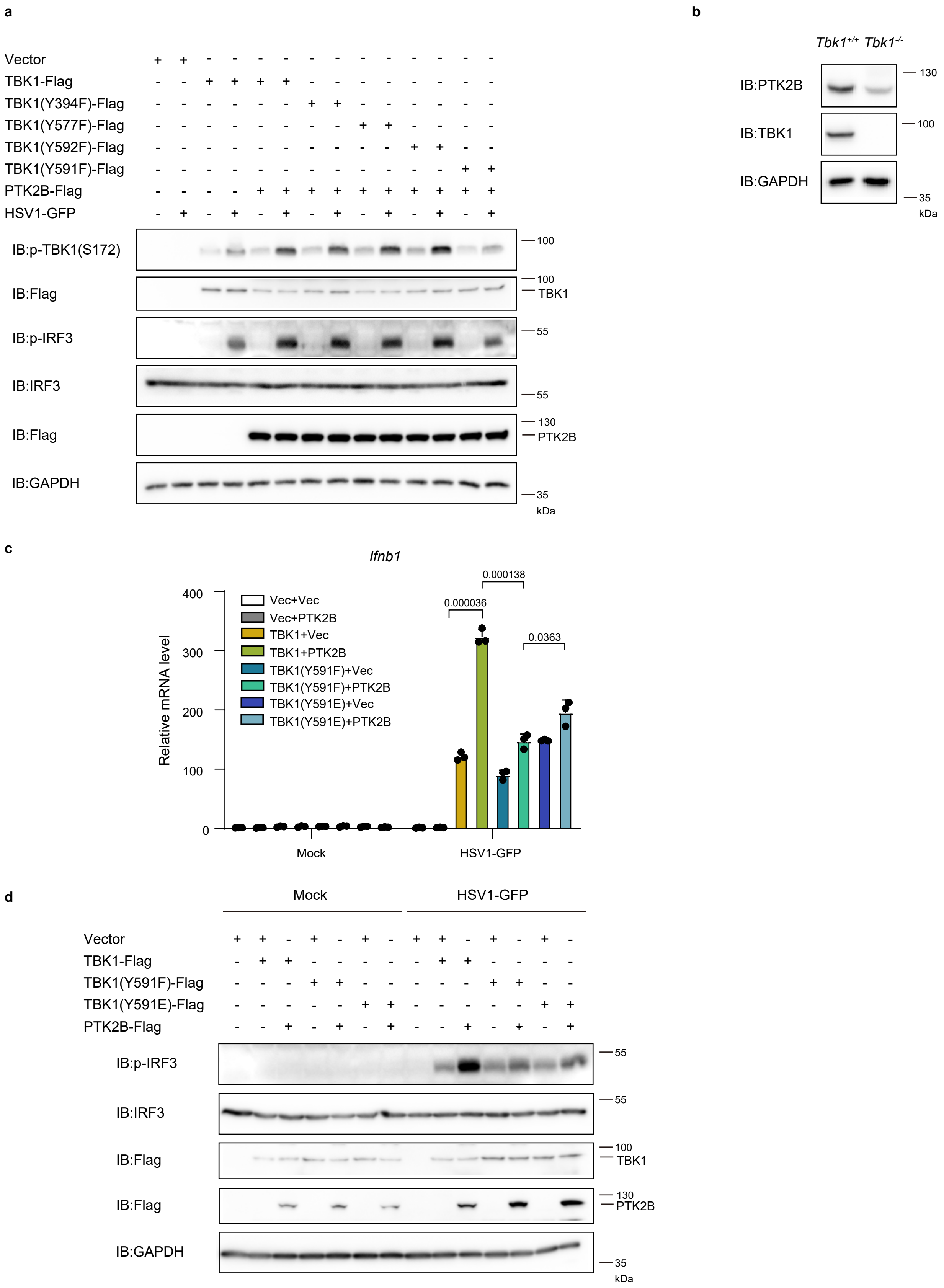


**Fig. S7 Phosphorylated Y591 of TBK1 by PTK2B plays an important role in regulating TBK1 activation**

(a) Immortalized TBK1-deficent MEF cells were co-expressed with PTK2B and wild-type TBK1 or its mutants, followed by infection with HSV1-GFP for 3 h. The cells were harvested for immunoblotting with the indicated antibodies.

(b) Immortalized TBK1-deficent and control MEFs were lysed for immunoblotting with the indicated antibodies.

(c, d) PTK2B and wild-type TBK1 or its mutants were co-expressed in TBK1-deficent MEF cells, and then the cells were infected with HSV1-GFP for 3 h. The cells were harvested for qPCR to measure the mRNA levels of *Ifnb1* (c) or for immunoblotting with the indicated antibodies (d).

Data are one representative of two independent experiments with similar results in (a, b ,d). Data shown in (c) are one representative experiment of three independent experiments (mean ± SD, n=3 independent samples), two-tailed Student's t-test. Source data in (a, b ,d) are provided in Fig. S13, source data in (c) are provided as a Source Data file.

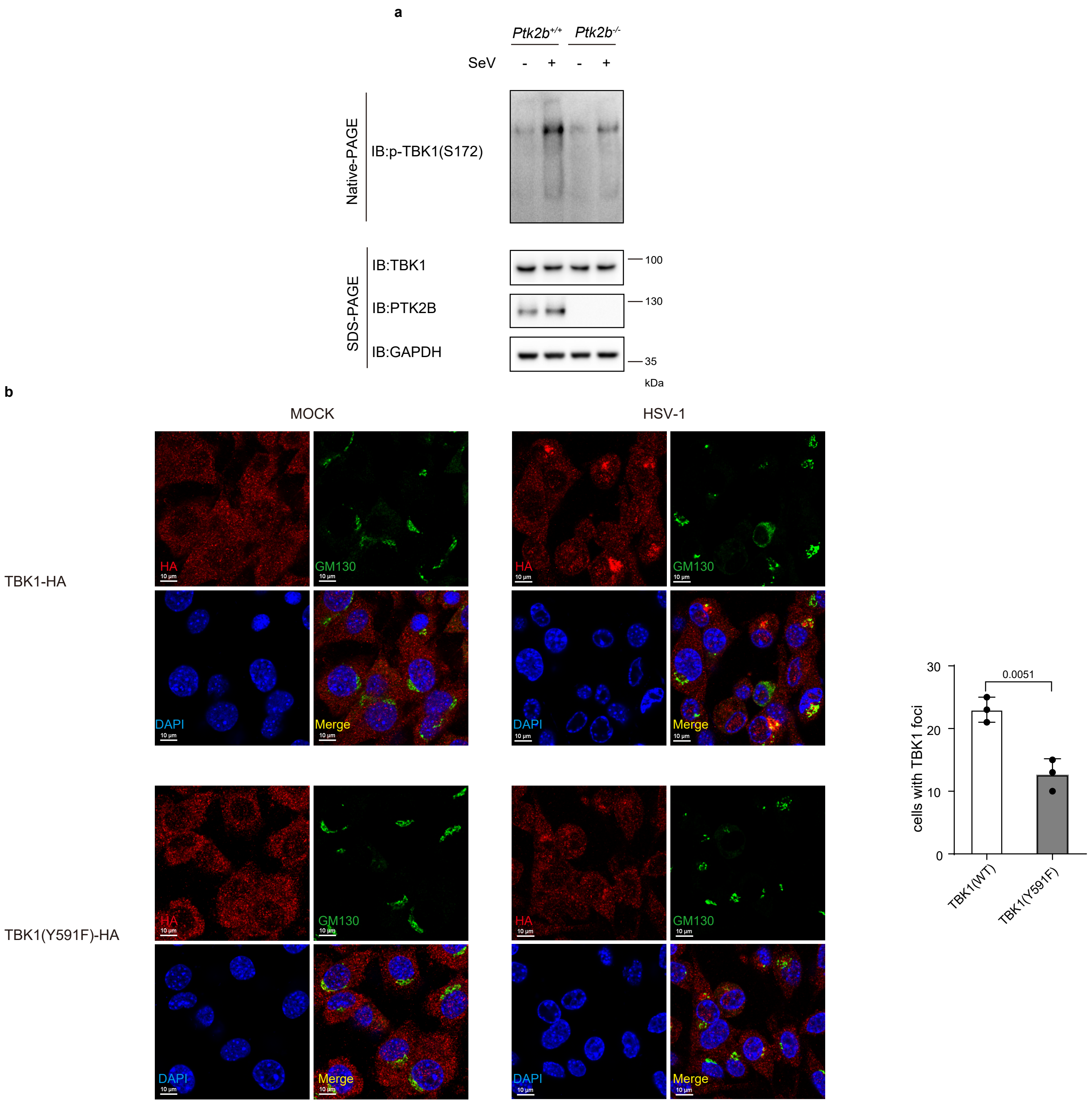


**Fig. S8 PTK2B mediates the oligomerization of TBK1**

(a) *Ptk2b*$^{+/+}$ and *Ptk2b*$^{-/-}$ Raw264.7 cells were mock infected or infected with SeV for 6 h. The cell lysates were resolved by Native-PAGE or SDS-PAGE, followed by immunoblotting.

(b) *Tbk1*$^{-/-}$ immortalized MEFs were co-expressed with PTK2B-Myc and TBK1-HA or its point mutant TBK1-Y591F-HA, and then mock infected or infected with HSV-1 for 6 h. The cells were stained with DAPI (blue), antibody to HA (red) and GM130 (Golgi marker, green), then imaged by confocal microscopy (left). Scale bars, 10 μm. The percentage of cells with TBK1-HA foci was quantified; n=105 cells from each group were analyzed (right).

Data are one representative of two independent experiments with similar results in (a). Data shown in (b) are from one representative experiment of three independent experiments, mean±SD, two-tailed Student's t-test. Source data in (a) are provided in Fig. S13, source data in (b) are provided as a Source Data file.

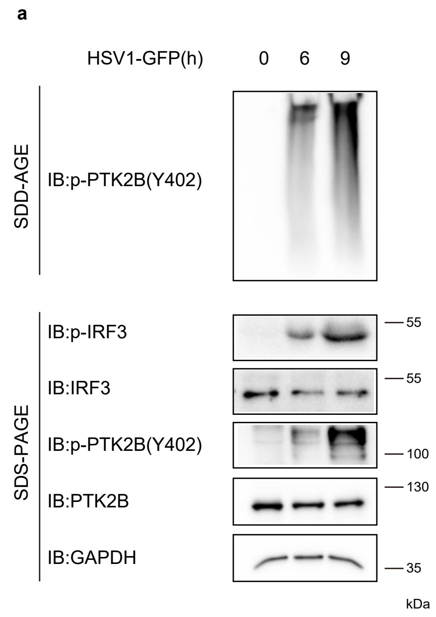


**Fig. S9 Phosphorylated PTK2B forms oligomerization upon HSV-1 infection**

(a) Raw264.7 cells were mock infected or infected with HSV1-GFP for the indicated times. The cell lysates were resolved by SDD-AGE or SDS-PAGE, followed by immunoblotting. Data shown in (a) are from one representative experiment of two independent experiments with similar results . Source data in (a) are provided in Fig. S13.

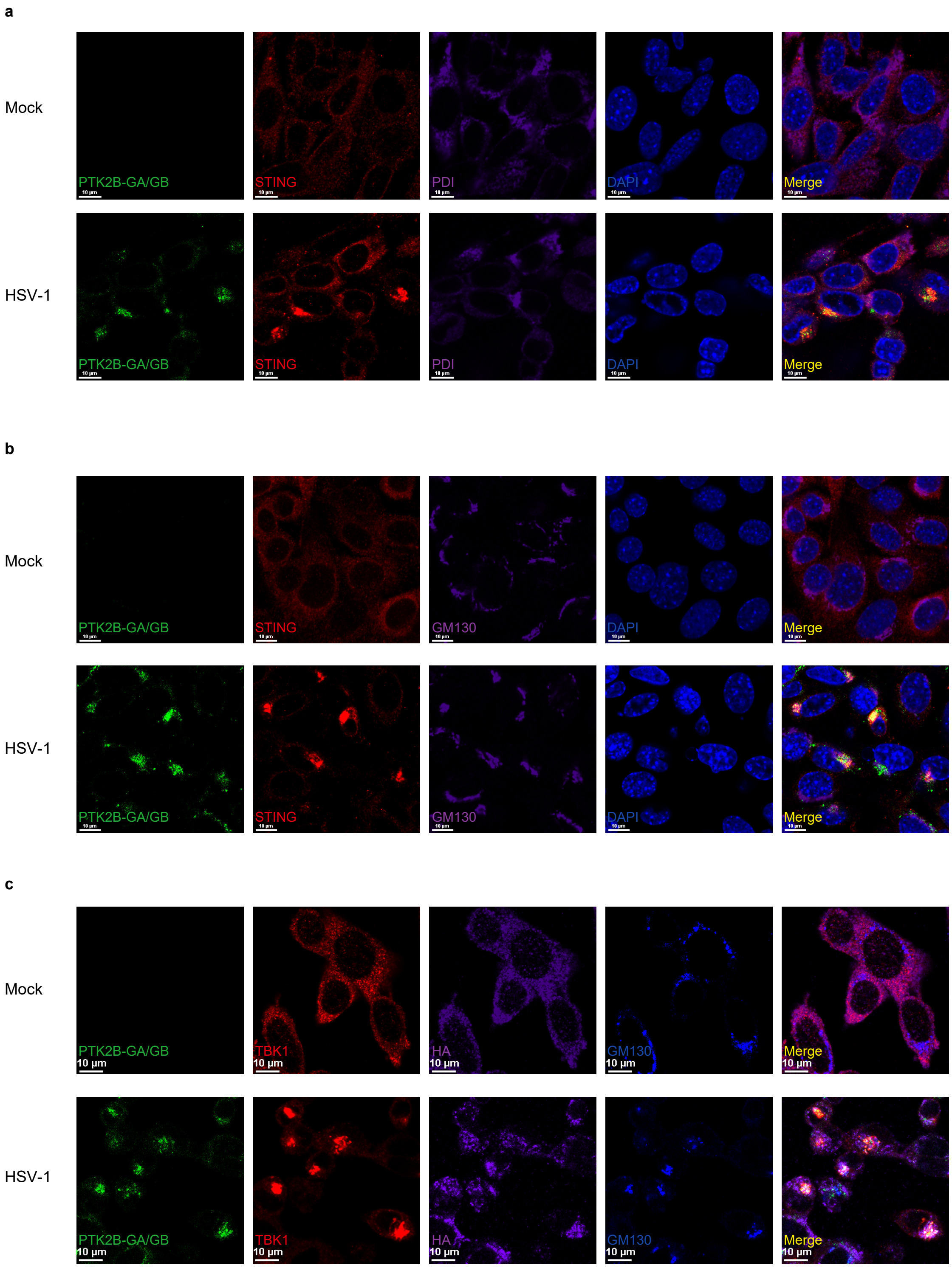


**Fig. S10 PTK2B, STING and TBK1 form granules on Golgi upon HSV-1 infection**

(a, b) Immortalized MEFs were co-expressed with PTK2B-GA, PTK2B-GB, followed by stimulation with or without HSV-1 for 6 h. The cells were fixed and stained with DAPI (blue), antibody to STING (red) and PDI (ER marker,purple)(a) or GM130 (Golgi marker, purple)(b)， then imaged by confocal microscopy. Scale bars, 10 μm.

(c) Immortalized MEFs were co-expressed with PTK2B-GA, PTK2B-GB and HA-STING, followed by stimulation with or without HSV-1 for 6 h. The cells were fixed and stained with GM130 (Golgi marker, blue), antibody to HA (purple) and TBK1 (red)， then imaged by confocal microscopy. Scale bars, 10 μm.

Data shown in (a-c) are from one representative experiment of three independent experiments. Source data are provided as a Source Data file.

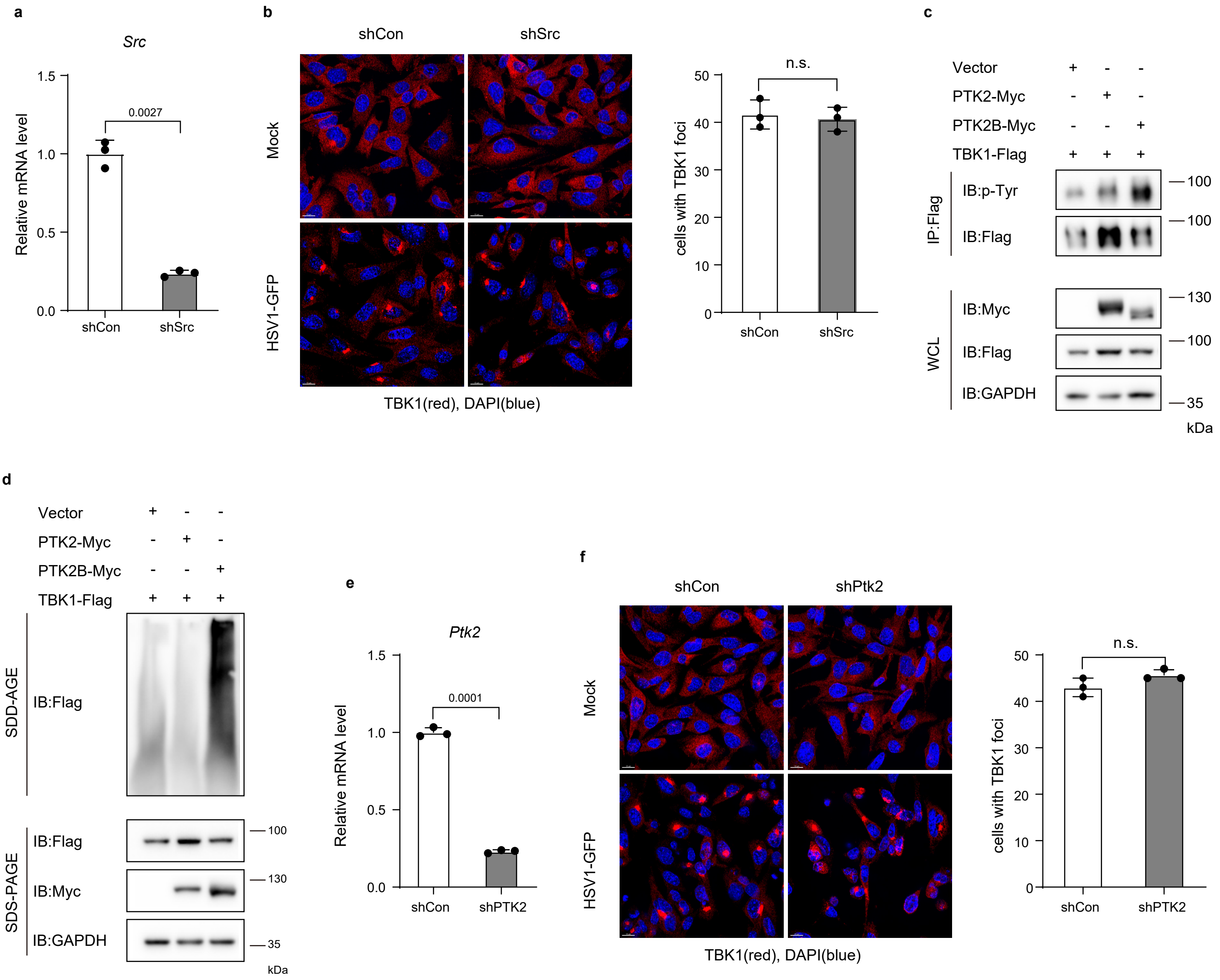


**Fig. S11 Src and PTK2 are not required for the formation of TBK1 oligomerization**

(a, b) Immortalized MEFs stably expressing shRNA targeting Src or negative control were infected with HSV1-GFP for 6 h, followed by qPCR (a) and immunofluorescence analysis of TBK1 (Red) and DAPI (b, left). The percentage of cells with TBK1 foci was quantified; n=105 cells from each group were analyzed (b, right).

(c) HEK293T cells were co-transfected with the indicated plasmids. IP assays were performed with anti-Flag M2 beads and analyzed by immunoblotting.

(d) HEK293T cells were co-transfected with the indicated plasmids. Cell lysates were resolved by SDD-AGE and SDS-PAGE, followed by immunoblotting with the indicated antibodies.

(e, f) Immortalized MEFs stably expressing shRNA targeting Ptk2 or negative control were infected with HSV1-GFP infection for 6 h, followed by qPCR (e) and immunofluorescence analysis of TBK1 (Red) and DAPI (f, left). The percentage of cells with TBK1 foci was quantified; n=105 cells from each group were analyzed (f, right).

Data shown in (a, b, e, f) are from one representative experiment of three independent experiments (mean ± SD, n=3 independent samples), two-tailed Student's t-test. Data are one representative of two independent experiments with similar results in (c, d). Source data in (a, b, e, f) are provided as a Source Data file, source data in (c, d) are provided in Fig. S13.

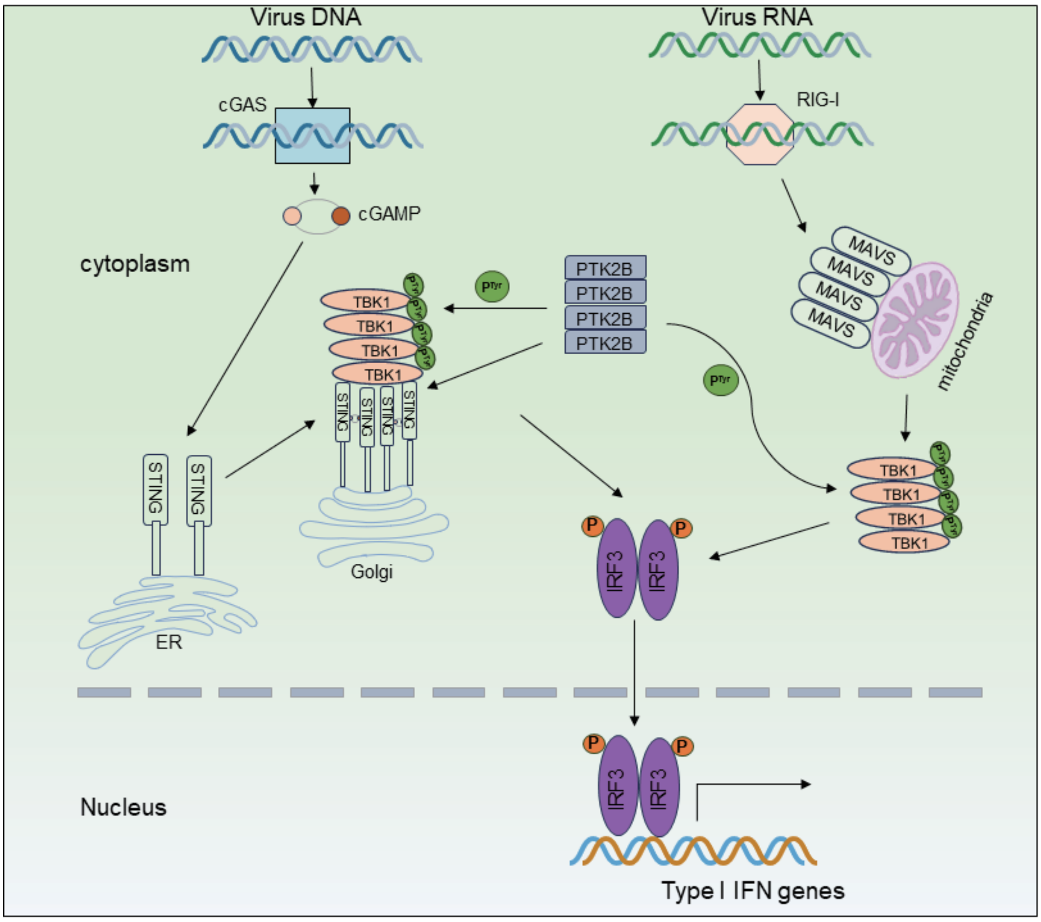


**Fig. S12 Schematic representation of PTK2B-mediated antiviral signaling**

Upon virus infection, PTK2B forms oligomerization, and enhances the oligomerization of TBK1 and STING, subsequently positively regulates STING-TBK1 activation to ensure efficient antiviral innate immune responses.

**Figure S1a**

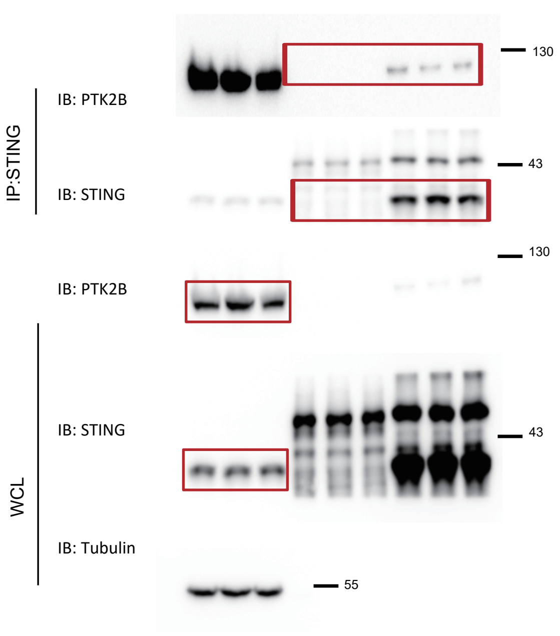


**Figure S1b**

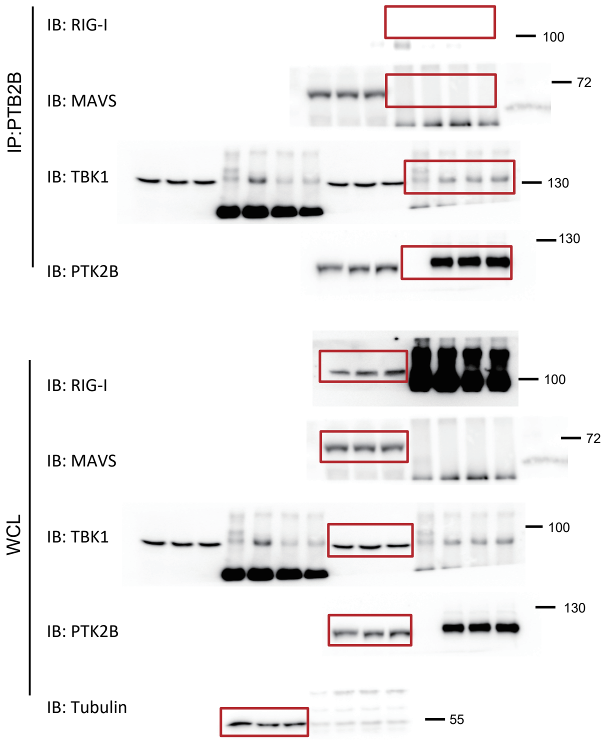


**Figure S1c**

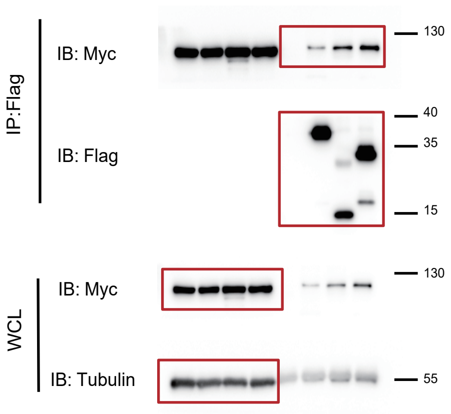


**Figure S1d**

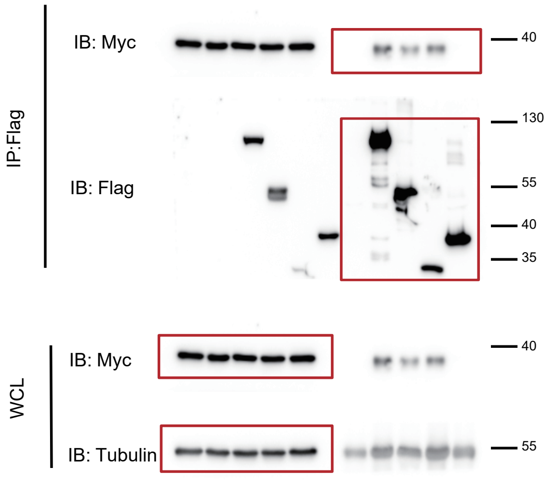


**Fig. S2g**

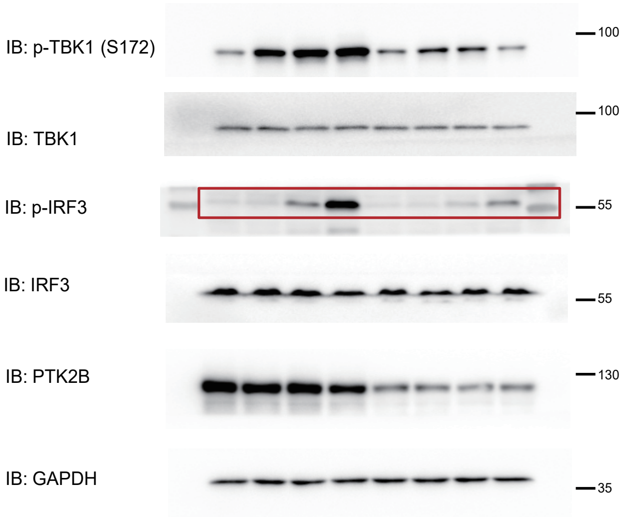


**Fig. S2h**

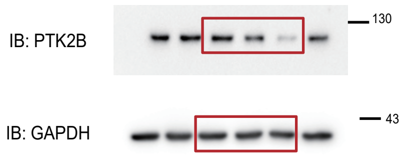


**Fig. S13 Original western blots in supplementary figures**

Panels corresponding to figures are indicated.

Fig. S3a

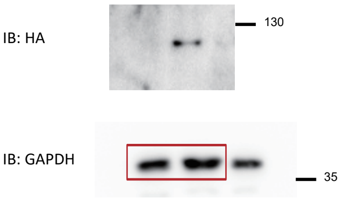


Fig. S3e

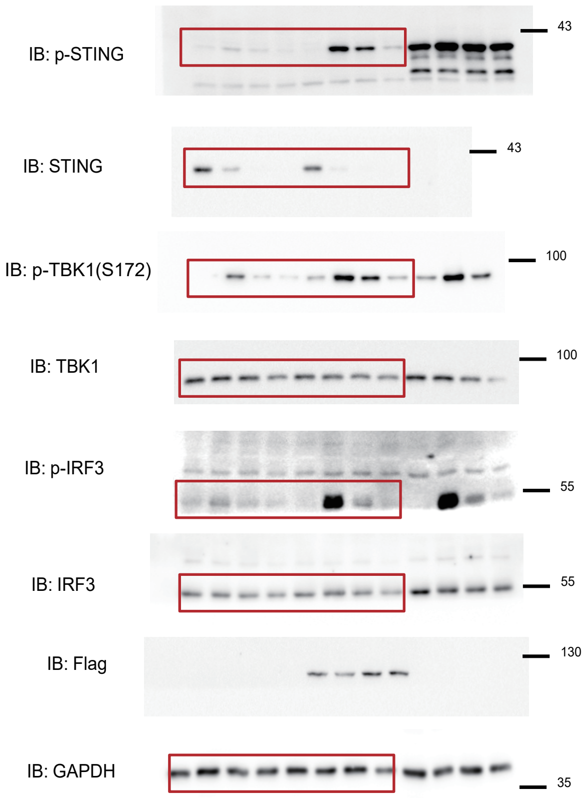


Fig. S3f

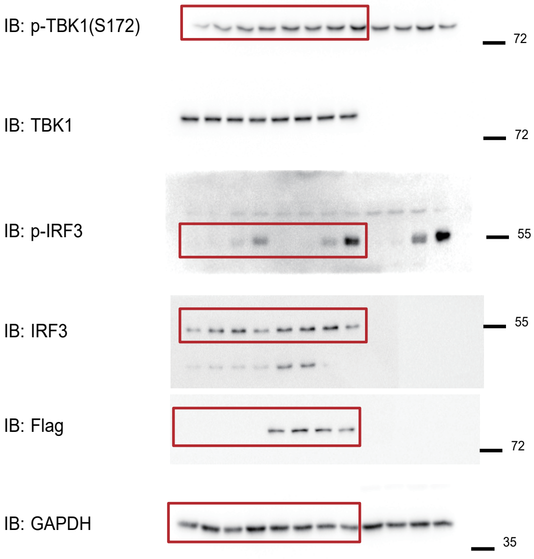


Fig. S4f

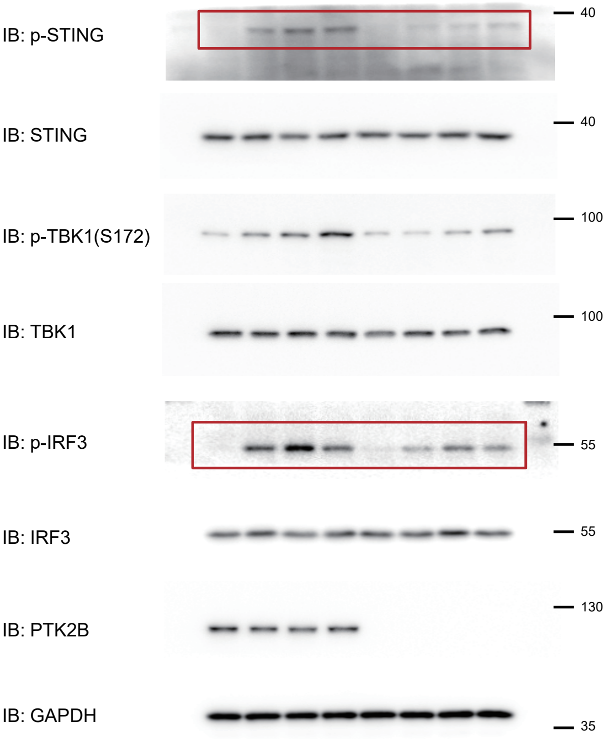


Fig. S3o

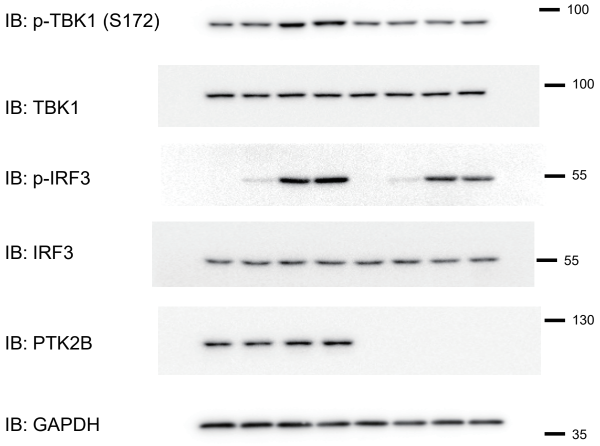


**Fig. S13 continued.**

Fig. S7a

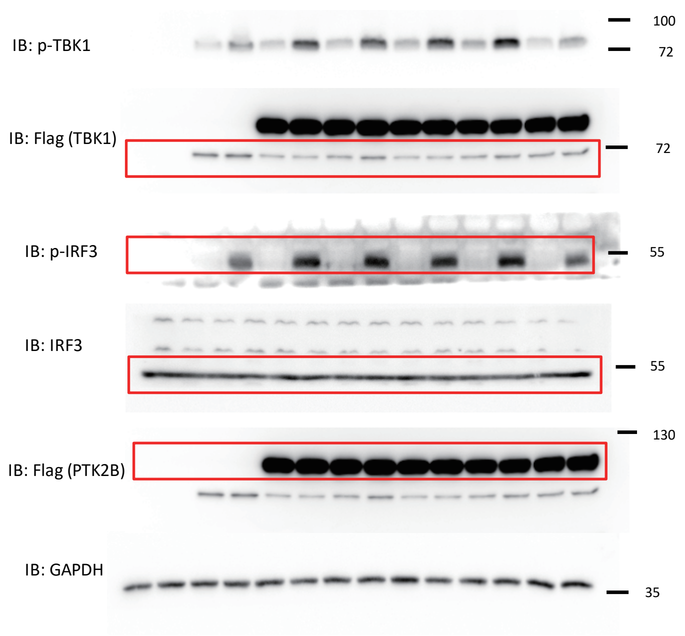


Fig. S7b

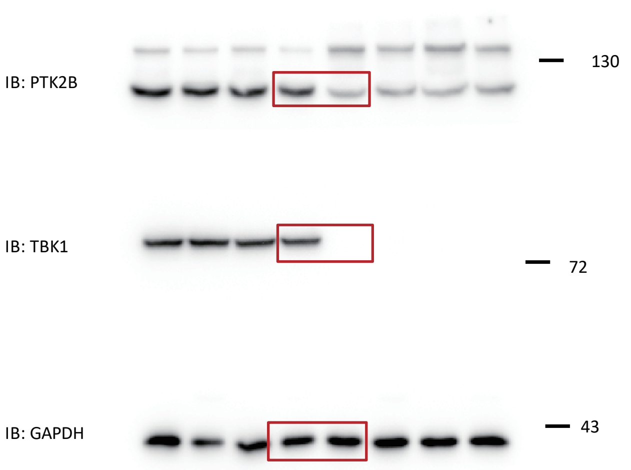


Fig. S7d

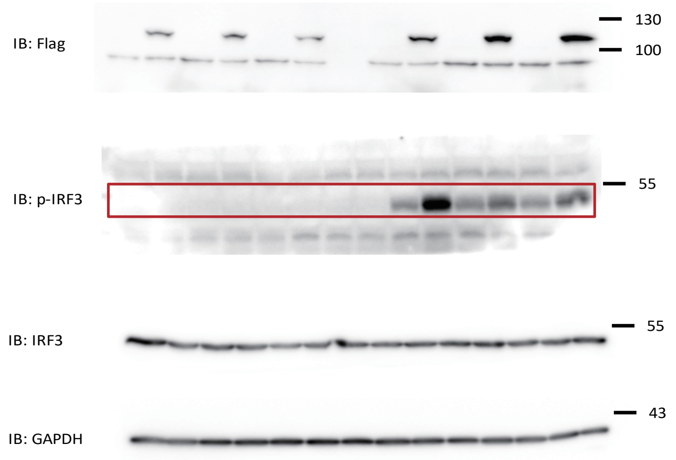


Fig. S8a

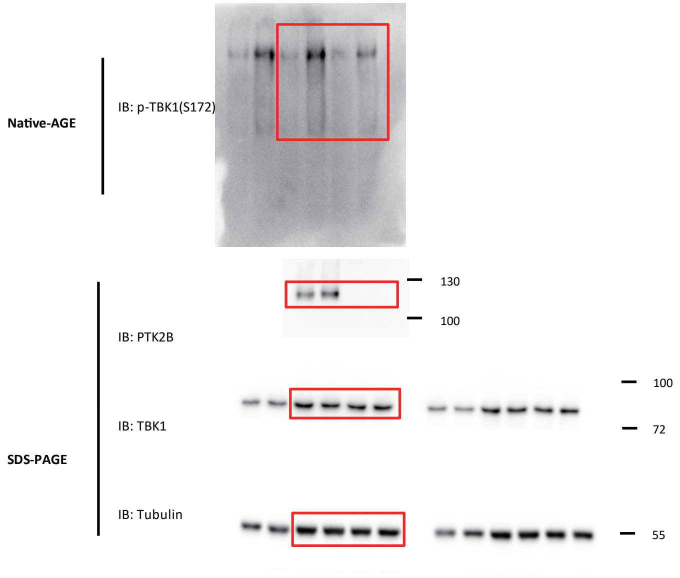


Fig. S9a

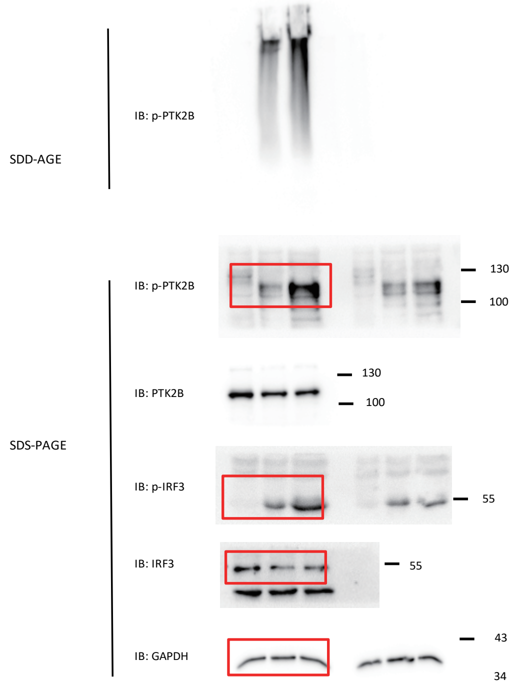


Fig. S13 continued.

**Fig. S11c**

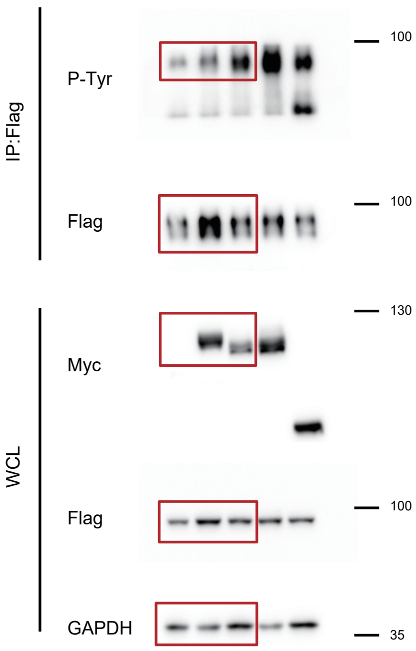


**Fig. S11d**

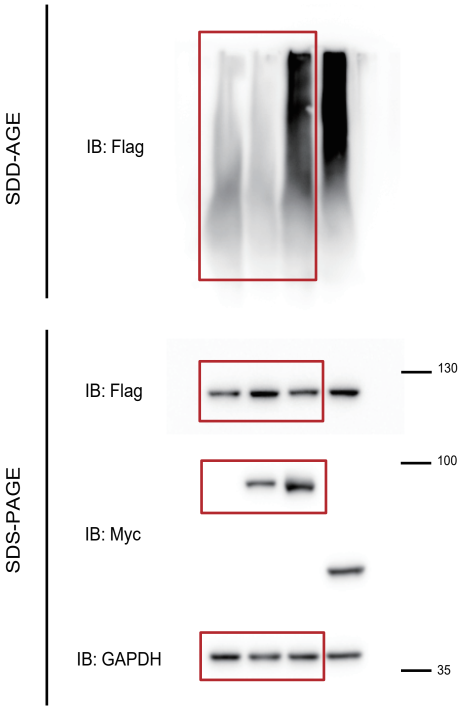


**Fig. S13 continued.**
