# Supplementary material for: PTK2B promotes TBK1 and STING oligomerization and enhances the STING-TBK1 signaling
Source: Nat Commun. 2023 Nov 21;14:7567. doi: 10.1038/s41467-023-43419-4 (PMC10663505; doi:10.1038/s41467-023-43419-4)
Supplement: Supplementary file 3 — Reporting Summary [file 41467_2023_43419_MOESM3_ESM.pdf]

nature portfolio

Corresponding author(s): Qinmiao Sun
Last updated by author(s): Oct 17, 2023

Reporting Summary

Nature Portfolio wishes to improve the reproducibility of the work that we publish. This form provides structure for consistency and transparency in reporting. For further information on Nature Portfolio policies, see our Editorial Policies and the Editorial Policy Checklist.

Statistics

For all statistical analyses, confirm that the following items are present in the figure legend, table legend, main text, or Methods section.

n/a	Confirmed	
☐	☒	The exact sample size (*n*) for each experimental group/condition, given as a discrete number and unit of measurement
☐	☒	A statement on whether measurements were taken from distinct samples or whether the same sample was measured repeatedly
☐	☒	The statistical test(s) used AND whether they are one- or two-sided<br>*Only common tests should be described solely by name; describe more complex techniques in the Methods section.*
☒	☐	A description of all covariates tested
☒	☐	A description of any assumptions or corrections, such as tests of normality and adjustment for multiple comparisons
☐	☒	A full description of the statistical parameters including central tendency (e.g. means) or other basic estimates (e.g. regression coefficient) AND variation (e.g. standard deviation) or associated estimates of uncertainty (e.g. confidence intervals)
☐	☒	For null hypothesis testing, the test statistic (e.g. *F*, *t*, *r*) with confidence intervals, effect sizes, degrees of freedom and *P* value noted<br>*Give P values as exact values whenever suitable.*
☒	☐	For Bayesian analysis, information on the choice of priors and Markov chain Monte Carlo settings
☒	☐	For hierarchical and complex designs, identification of the appropriate level for tests and full reporting of outcomes
☐	☒	Estimates of effect sizes (e.g. Cohen's *d*, Pearson's *r*), indicating how they were calculated

Our web collection on statistics for biologists contains articles on many of the points above.

Software and code

Policy information about availability of computer code

Data collection	The software used for collecting RT-qPCR data was Bio-Rad CFX Maestro 2.0. The software used for FACS data was BD FACSDiva v8.0.3. Imaging used an Andor Dragonfly 505 laser scanning system and Nikon A1 confocal microscope.The software used for collecting western blots data was FLICapture (version 1.02).The software used for collecting elisa data was OPTIMA (version 2.20).LC-MS/MS assays using an Orbitrap Elite mass spectrometer (Thermo Fisher Scientific) and nanoflow Easy-nLC (Thermo Scientific) and an Orbitrap hybrid mass spectrometer (Orbitrap Exploris 480, Thermo Scientific).
Data analysis	FlowJo (version 10.0.7)<br>Image J (version 1.8.0)<br>GraphPad Prism software (version 8.0.2)<br>GIS 1D(version 4.2)<br>Imaris 9.5.1 software<br>NIS-Elements AR analysis 5.20.00 software<br>NIS-Elements F analysis 3.22.00 software<br>Thermo Proteome Discovery (version2.3)<br>MaxQuant software version (1.6.4.0)

For manuscripts utilizing custom algorithms or software that are central to the research but not yet described in published literature, software must be made available to editors and reviewers. We strongly encourage code deposition in a community repository (e.g. GitHub). See the Nature Portfolio guidelines for submitting code & software for further information.

Data

Policy information about availability of data

All manuscripts must include a data availability statement. This statement should provide the following information, where applicable:

- Accession codes, unique identifiers, or web links for publicly available datasets
- A description of any restrictions on data availability
- For clinical datasets or third party data, please ensure that the statement adheres to our policy

The authors declare that the data supporting the findings of this study are available within the article and its Supplementary Information file.The mass spectrometry proteomics data have been deposited to the ProteomeXchange Consortium (http://proteomecentral.proteomexchange.org) via the iProX partner repository with the dataset identifier PXD045961. Mass spectra were searched against the Uniprot Mus musculus database 2022.11.16.Source data are provided with this paper.

Research involving human participants, their data, or biological material

Policy information about studies with human participants or human data. See also policy information about sex, gender (identity/presentation), and sexual orientation and race, ethnicity and racism.

Reporting on sex and gender	Not applicable
Reporting on race, ethnicity, or other socially relevant groupings	Not applicable
Population characteristics	Not applicable
Recruitment	Not applicable
Ethics oversight	Not applicable

Note that full information on the approval of the study protocol must also be provided in the manuscript.

Field-specific reporting

Please select the one below that is the best fit for your research. If you are not sure, read the appropriate sections before making your selection.

☒ Life sciences ☐ Behavioural & social sciences ☐ Ecological, evolutionary & environmental sciences

For a reference copy of the document with all sections, see nature.com/documents/nr-reporting-summary-flat.pdf

Life sciences study design

All studies must disclose on these points even when the disclosure is negative.

Sample size	The sample size for in vivo and in vitro experiments was determined on the basis of previous publications and our experience. (PMID: 35322803,PMID: 30510222).
Data exclusions	No data were excluded from the studies.
Replication	All Experiments were repeated at least two times to ensure reproducibility of data. Please refer to figure legend for details. All attempts are successful.
Randomization	For the mouse experiments, animal were randomly grouped before treatment.
Blinding	No blinding is applicable to the study, as all cell and animal treatment conditions and groups are clear to the researchers.

Reporting for specific materials, systems and methods

We require information from authors about some types of materials, experimental systems and methods used in many studies. Here, indicate whether each material, system or method listed is relevant to your study. If you are not sure if a list item applies to your research, read the appropriate section before selecting a response.

Materials & experimental systems

n/a	Involved in the study
	x Antibodies
	x Eukaryotic cell lines
x	Palaeontology and archaeology
	x Animals and other organisms
x	Clinical data
x	Dual use research of concern
x	Plants

Methods

n/a	Involved in the study
x	ChIP-seq
	x Flow cytometry
x	MRI-based neuroimaging

Antibodies

Antibodies used

Primary antibodies:
Rabbit anti-STING (Cell Signaling Technology, Cat# 50494,1:1000 for WB)
Rabbit anti-human phospho-STING (Cell Signaling Technology,Cat# 19781,1:1000 for WB,1:500 for Immunoprecipitation)
Rabbit anti-mouse phospho-STING (Cell Signaling Technology,Cat# 72971,1:1000 for WB)
Rabbit anti-IRF3 (Cell Signaling Technology,Cat#4302,1:500 for WB)
Rabbit anti-phospho-IRF3 (Cell Signaling Technology,Cat# 4947,1:500 for WB)
Rabbit anti-phospho-TBK1 (Cell Signaling Technology,Cat# 5483,1:1000 for WB)
mouse anti-PTK2B antibodies (Cell Signaling Technology,Cat# 3480,1:1000 for WB)
Rabbit anti-phospho-PTK2B antibodies (Cell Signaling Technology,Cat# 3291,1:1000 for WB)
mouse anti-phospho-Tyrosine antibodies (Cell Signaling Technology,Cat# 9416,1:1000 for WB)
mouse anti-PDI antibodies(Cell Signaling Technology,Cat# 45596,1:1000 for WB)
Rabbit anti-TBK1 (Abcam,Cat# ab40676,1:1000 for WB,1:500 for immunostaining)
Rabbit anti-PTK2B (Abcam,Cat# ab226798,1:1000 for WB,1:500 for immunostaining ,1:500 for Immunoprecipitation)
Rabbit anti-TBK1 (Santa Cruz Biotechnology,Cat# sc-52957,1:1000 for WB,1:500 for immunostaining)
Rabbit anti-STING for immunostaining (Proteintech,Cat# 19851-1-AP,1:500)
Mouse anti-glyceraldehyde-3-phosphate dehydrogenase (GAPDH) (Sungene Biotechnology,Cat# KM9002,1:5000 for WB)
mouse anti-α-Tubulin (Sungene Biotechnology,Cat# KM9007,1:5000 for WB)
Mouse anti-Flag (MBL,Cat# M185-3,1:2000 for WB,1:500 for immunostaining)
Rabbit anti-HA (MBL,Cat# 561,1:2000 for WB,1:500 for immunostaining)
Rabbit anti-Myc (MBL,Cat# 562,1:2000 for WB,1:500 for immunostaining)
Mouse anti-GST antibody (OriGene Technologies,Cat# TA150101,1:1000 for WB)
Rabbit anti-His antibody (Biodragron,Cat# B1023,1:1000 for WB)
Specific Rabbit antibody for the phosphorylation of TBK1 at tyrosine 591 was generated in ABclonal Technology(1:1000 for WB).
Rabbit anti-Rig-I (Cell Signaling Technology,Cat# 3743S,1:500 for WB),
Mouse anti-GM130(BD Biosciences,Cat#610822,1:500 for immunostaining)
WB secondary antibodies:
Goat anti-Rabbit IgG HL(HRP)(Abcam,Cat# AB205718,1:2000 for WB)
Goat anti-Mouse IgG HL(HRP)(Abcam,Cat# AB205719,1:2000 for WB)
immunofluorescent staining secondary antibodies:
Alexa Fluor 555 goat anti-mouse IgG(H+L)(Invitrogen,A21422,1:2000)
Flow cytometry antibodies:
Rat anti-mouse/human CD11b bralliant violet 510(Biolegend,Cat# 101263,1:50)
Rat anti-mouse F4/80 APC(Biolegend,Cat# 123115,1:125)
Rat anti-mouse Ly-6G PE(Biolegend,Cat# 127607,1:125)
American hamster anti-mouse CD11c (Biolegend,Cat# 117305,1:500)

Validation

All the antibodies were from commercial sources and verified by the manufactures. Validation statements and experiments can be obtained from the following websites:
Rabbit anti-STING (https://www.cellsignal.cn/products/primary-antibodies/sting-d1v5l-rabbit-mab/50494)
Rabbit anti-human phospho-STING (https://www.cellsignal.cn/products/primary-antibodies/phospho-sting-ser366-d7c3s-rabbit-mab/19781)
Rabbit anti-mouse phospho-STING (https://www.cellsignal.cn/products/primary-antibodies/phospho-sting-ser365-d8f4w-rabbit-mab/72971)
Rabbit anti-IRF3 (https://www.cellsignal.cn/products/primary-antibodies/irf-3-d83b9-rabbit-mab/4302)
Rabbit anti-phospho-IRF3 (https://www.cellsignal.cn/products/primary-antibodies/phospho-irf-3-ser396-4d4g-rabbit-mab/4947)
Rabbit anti-TBK1 (https://www.cellsignal.cn/products/primary-antibodies/phospho-tbk1-nak-ser172-d52c2-xp-rabbit-mab/5483)
mouse anti-PTK2B antibodies (https://www.cellsignal.cn/products/primary-antibodies/pyk2-5e2-mouse-mab/3480)
Rabbit anti-phospho-PTK2B antibodies (https://www.cellsignal.cn/products/primary-antibodies/phospho-pyk2-tyr402-antibody/3291)
mouse anti-phospho-Tyrosine antibodies (https://www.cellsignal.cn/products/primary-antibodies/phospho-tyrosine-mouse-mab-p-tyr-102/9416)
mouse anti-PDI antibodies(https://www.cellsignal.cn/products/primary-antibodies/pdi-e7o2r-mouse-mab/45596)
Rabbit anti-TBK1 (https://www.abcam.cn/products/primary-antibodies/naktbk1-antibody-ep611y-ab40676.html)

Rabbit anti-PTK2B (https://www.abcam.cn/products/primary-antibodies/pyk2-antibody-ab226798.html)
Rabbit anti-TBK1 (https://www.scbt.com/p/tbk1-antibody-108a429?requestFrom=search)
Rabbit anti-STING(https://www.ptgcn.com/products/TMEM173-Antibody-19851-1-AP.htm)
Mouse anti-glyceraldehyde-3-phosphate dehydrogenase (GAPDH) (http://www.sungenebiotech.com/index.php?m=Product&a=product_xq&catid=2&proid=53&prid=290&pid=720&id=1557)
mouse anti-α-Tubulin (http://www.sungenebiotech.com/index.php?m=Product&a=product_xq&catid=2&proid=53&prid=291&pid=723&id=1566)
Mouse anti-Flag (https://www.mblbio.com/bio/g/dtl/A/?pcd=M185-3L)
Rabbit anti-HA (https://www.mblbio.com/bio/g/dtl/A/?pcd=561)
Rabbit anti-Myc (https://www.mblbio.com/bio/g/dtl/A/?pcd=562)
Mouse anti-GST antibody (https://www.origene.com/catalog/antibodies/tag-antibodies/ta150101/clone-oti2a4-anti-gst-mouse-monoclonal-antibody)
Rabbit anti-His antibody (https://www.biodragon.cn/plus/view.php?aid=74632)
Rabbit anti-Rig-I (https://www.cellsignal.cn/products/primary-antibodies/rig-i-d14g6-rabbit-mab/3743)
Mouse anti-GM130(https://www.bdbiosciences.com/en-us/products/reagents/microscopy-imaging-reagents/immunofluorescence-reagents/purified-mouse-anti-gm130.610822)

Eukaryotic cell lines

Policy information about cell lines and Sex and Gender in Research

Cell line source(s)	HEK293T, HeLa, THP-1, Vero and RAW264.7 cells were kindly provided by Stem Cell Bank, Chinese Academy of Science from the Shanghai Cell Bank of the Chinese Academy of Sciences (https://www.cellbank.org.cn/). TBK1-knockout MEFs were kindly provided by Zhengfan Jiang's lab from Peking University.
Authentication	We didn't authenticate cell lines.
Mycoplasma contamination	The cell line was not tested for mycoplasma.
Commonly misidentified lines (See ICLAC register)	No commonly misidentified cell lines were used.

Animals and other research organisms

Policy information about studies involving animals; ARRIVE guidelines recommended for reporting animal research, and Sex and Gender in Research

Laboratory animals	Ptk2b heterozygous mice were obtained from Shanghai Model Organisms Center(C576j).Both PTK2B knockout mice and wild-type mice were bred in our own laboratory. The experimental mice were all 10-12 weeks old and the mice in the same experiment were the same age. Mice were housed in groups of up to 5 mice/cage at 18 -24 °C ambient temperatures with 40-60% humidity. Mice were maintained on a 12 hour light/ dark cycle 6 am to 6 pm. Food and water were available ad libitum.
Wild animals	Not relevant to this study.
Reporting on sex	The results of this study were not affected by the sex of the mice.
Field-collected samples	This study did not involve samples collected from field.
Ethics oversight	The protocols for animal studies were approved by the Committee on the Ethics of Animal Experiments of the Institute of Zoology,Chinese Academy of Sciences (Beijing, China) (approval number:IOZ2021009).

Note that full information on the approval of the study protocol must also be provided in the manuscript.

Flow Cytometry

Plots

Confirm that:

- [x] The axis labels state the marker and fluorochrome used (e.g. CD4-FITC).
- [x] The axis scales are clearly visible. Include numbers along axes only for bottom left plot of group (a 'group' is an analysis of identical markers).
- [x] All plots are contour plots with outliers or pseudocolor plots.
- [x] A numerical value for number of cells or percentage (with statistics) is provided.

Methodology

Sample preparation	The lung tissue of mice was digested by collagenase D, then 5*10^5/ml cells were stained with corresponding antibodies and followed by flow analysis.

Instrument	BD Influx Fortessa
Software	BD FACSDiva (v8.0.3)
Cell population abundance	CD11b-BV510-positive, F4/80-APC-positive, Ly-6G-PE -positive and CD11c-FITC-positive cells were sorted
Gating strategy	Macrophages (CD11b-BV510-positive, F4/80-APC-positive);Neutrophils(CD11b-BV510-positive, Ly-6G-PE -positive); DCs (CD11c-FITC-positive)

☒ Tick this box to confirm that a figure exemplifying the gating strategy is provided in the Supplementary Information.
